# Neural Correlates of Smartphone Dependence in Adolescents

**DOI:** 10.3389/fnhum.2020.564629

**Published:** 2020-10-07

**Authors:** Olga Tymofiyeva, Justin P. Yuan, Roma Kidambi, Chiung-Yu Huang, Eva Henje, Mark L. Rubinstein, Namasvi Jariwala, Jeffrey E. Max, Tony T. Yang, Duan Xu

**Affiliations:** ^1^Department of Radiology & Biomedical Imaging, University of California, San Francisco, San Francisco, CA, United States; ^2^Department of Psychology, Stanford University, Stanford, CA, United States; ^3^Department of Epidemiology and Biostatistics, University of California, San Francisco, San Francisco, CA, United States; ^4^Department of Psychiatry and Behavioral Sciences, The Langley Porter Psychiatric Institute, Division of Child and Adolescent Psychiatry, Weill Institute for Neurosciences, University of California, San Francisco, San Francisco, CA, United States; ^5^Department of Clinical Science, Child and Adolescent Psychiatry, Umeå University, Umeå, Sweden; ^6^Department of Pediatrics, University of California, San Francisco, San Francisco, CA, United States; ^7^Department of Psychiatry, University of California, San Diego, Rady Children’s Hospital-San Diego, San Diego, CA, United States

**Keywords:** smartphone dependence, adolescent, brain connectivity, depression, sleep

## Abstract

Increases in depressive and suicide-related symptoms among United States adolescents have been recently linked to increased use of smartphones. Understanding of the brain mechanisms that underlie the potential smartphone dependence may help develop interventions to address this important problem. In this exploratory study, we investigated the neural mechanisms underlying potential smartphone dependence in a sample of 19 adolescent volunteers who completed self-assessments of their smartphone dependence, depressive symptoms, and sleep problems. All 19 adolescents underwent diffusion MRI that allowed for assessment of white matter structural connectivity within the framework of connectomics. Based on previous literature on the neurobiology of addiction, we hypothesized a disruption of network centrality of three nodes in the mesolimbic network: Nucleus Accumbens, anterior cingulate cortex, and amygdala. Our results showed positive correlations between the node centrality of the right amygdala and self-reported smartphone dependence, between smartphone dependence and sleep problems, and between sleep problems and depressive symptoms. A higher phone dependence was observed in females compared to males. Supported by these results, we propose a model of how smartphone dependence can be linked to aberrations in brain networks, sex, sleep disturbances, and depression in adolescents.

## Introduction

A recent study reported alarming increases in depressive symptoms, suicide-related outcomes, and suicide rates among United States adolescents and links to increased new media screen time (including social media and electronic devices such as smartphones) (Twenge et al., 2018). While bidirectional causality cannot be excluded, evidence supports the model in which increased screen time leads to increased mental health problems, especially in females (Twenge et al., 2018). One potential mediating link between excessive smartphone use and mental health problems are the sleep problems caused by excessive screen time (Van den Bulck, 2007; Demirci et al., 2015), as sleep has been often causally implicated in depression (Baglioni et al., 2011).

Internet-enabled smartphones give us unprecedented freedom to access information and communicate but, at the same time, they can lead to dependence (Roberts et al., 2014; Kuss et al., 2018). Behavioral dependence is understood as a repetitive occurrence of impulsive behaviors, which can lead to harmful consequences (Lejoyeux et al., 2000). There is, however, a large gap in our understanding of the neural mechanisms underlying potential smartphone dependence, which complicates the development of suitable recommendations and interventions targeting excessive phone use. While we use the term “dependence” here, excessive use of a smartphone may meet the definition of a “behavioral addiction,” as it can potentially lead to distress or impairment in major areas of life functioning (APA, 2013). The Diagnostic and Statistical Manual of Mental Disorders (DSM-5) currently only considers gambling disorder to be a behavioral addiction, and Internet gaming disorder (IGD) – a “condition for further study” (APA, 2013). Recently (summer 2018), the World Health Organization (WHO) included gaming disorder into the International Statistical Classification of Diseases (ICD) (WHO, 2018). The following three diagnostic requirements need to be met and last over a period of at least 12 months to diagnose someone with gaming disorder: the person’s control over gaming is impaired, gaming is strongly preferred over other life interests and daily activities, and the person does not stop gaming even despite negative consequences impairing personal, social, educational, occupational, or other important areas of life (WHO, 2018).

In contrast to gambling and Internet gaming, smartphone use may span various activities: messaging/texting, social networking, gaming, watching videos, etc., All modes of smartphone use, however, have unifying characteristics, such as pervasive stimulation due to the constant personal accessibility of the phone and salience of the sensory stimuli. It is therefore essential to study smartphone use as a separate category of behaviors with the potential to cause addiction. Previous findings in the field of neurobiology of addiction can guide the hypotheses in studying smartphone dependence.

The mesolimbic brain circuit – in particular, the Nucleus Accumbens (NAcc), anterior cingulate cortex (ACC), and amygdala – is known to play a central role in both substance-based (Kühn and Gallinat, 2011) and behavioral addictions (Voon et al., 2014). The NAcc, where mesolimbic dopamine is released, is the neural substrate of the feeling of reward (Robinson and Berridge, 2001). The amygdala links the characteristics of the environmental stimuli to their affective or emotional attributes and thus to the reward system (Bechara, 2005). In addicted individuals, the amygdala becomes over-sensitized due to repetition of a rewarding behavior and becomes overresponsive to reward – the incentive salience attribution process described as “wanting” (Robinson and Berridge, 2001). Together, the amygdala and NAcc form the “impulsive system,” underlying automatic, habitual, impulsive behaviors (Noël et al., 2013). At the same time, ACC is part of the self-control, “reflective” system that inhibits responses to impulses (Noël et al., 2013).

It is being increasingly recognized that neural substrates of psychiatric and neurologic disorders (or any complex behavior in general) are not limited to isolated brain regions but include anatomical white matter connections between different regions (Griffa et al., 2013). In alignment with this view, MRI connectomics treats the brain as a complex network composed of *nodes* and *edges* (Hagmann et al., 2010a). All networks can be characterized using graph theory in terms of global and local properties, e.g., network *centrality* of a node (the sum of connection weights between a brain region and the rest of the brain) (Hagmann et al., 2010a). MRI connectomics has been applied to both the adult and developing brain (Hagmann et al., 2010b; Tymofiyeva et al., 2012, 2013). This framework has also been applied to study the neural signature of adult depression (Korgaonkar et al., 2014), as well as adolescent depression (Tymofiyeva et al., 2017, 2018).

Understanding the disruptions of anatomical brain networks associated with smartphone dependence in adolescents could help us develop recommendations and interventions for reducing adolescent addiction to smartphones and potentially preventing adolescent depression and suicide. Two previous studies investigated white matter microstructure in young adults with mobile phone dependence (Wang et al., 2016; Hu et al., 2017). Specifically, tract-based spatial statistics (TBSS) conducted in these two studies revealed microstructural aberrations in bilateral hippocampal cingulum bundle fibers (Wang et al., 2016) and in superior longitudinal fasciculus (SLF), superior corona radiata (SCR), internal capsule, external capsule, sagittal stratum, fornix/stria terminalis and midbrain structures (Hu et al., 2017). Our goal was to extend this research effort to analyzing neural correlates of smartphone dependence in the context of networks in the adolescent brain, as developmental processes can play a significant role in smartphone dependence.

To fill this significant knowledge gap, the aim of this exploratory study was to assess the relationship between smartphone dependence in adolescents and their structural brain networks mapped using diffusion MRI. Based on previous literature on neurobiology of addiction (Robinson and Berridge, 2001; Bechara, 2005; Kühn and Gallinat, 2011; Voon et al., 2014), we hypothesized a disruption of network centrality of three nodes of the brain network: NAcc (segmented as part of caudate), ACC, and amygdala. While testing this hypothesis, we accounted for the role of sex in smartphone dependence, as multiple studies have suggested that females may have higher smartphone dependence (Punamäki et al., 2007; Twenge et al., 2018).

We additionally explored behavioral correlations between smartphone dependence and sleep problems and that between sleep problems and depressive symptoms. Based on the reviewed literature, we hypothesized positive correlations in both cases (Van den Bulck, 2007; Baglioni et al., 2011; Demirci et al., 2015).

## Materials and Methods

### Participants

The study was approved by the Institutional Review Board (IRB) of the University of California, San Francisco and all participants in the study provided written informed assent and their parent(s) or legal guardian(s) provided written informed consent in accordance with the Declaration of Helsinki. A community sample of 19 adolescent volunteers (mean ± standard deviation, 16.3 ± 1.2 years; range, 14–18 years; 8 females and 11 males) participated in this neuroimaging study.

### Behavioral Self-Reported Measures

The following behavioral self-reported measures were acquired. (1) The Smartphone Addiction Scale - Short Version (SAS-SV) is a valid and reliable self-assessment measure of the degree of smartphone addiction in teenagers (Kwon et al., 2013). The following six factors are considered in the questionnaire: daily life disturbance, positive anticipation, withdrawal, cyberspace-oriented relationship, overuse, and tolerance. (2) The Insomnia Severity Index (ISI) is a commonly used, valid and reliable self-assessment scale for global insomnia symptoms with scores ranging from 0 to 28 (Bastien et al., 2001; Chung et al., 2011). (3) The Reynolds Adolescent Depression Scale-2 (RADS-2) (Reynolds, 2002) is a widely used, valid and reliable self-report measure of adolescent depression that provides scores on four subscales that evaluate specific domains of depressive symptoms in adolescents: Anhedonia, Dysphoric Mood, Negative Self-Evaluation, and Somatic Complaints (Reynolds and Mazza, 1998; March et al., 2006; Osman et al., 2010).

### MRI Data Acquisition and Network Construction

Each adolescent underwent an MRI scan at a 3T General Electric MR750 MRI scanner and NOVA Medical 32-channel head coil. The scan included a standard T1-weighted sequence and a spin-echo echo-planar-imaging (EPI) diffusion tensor imaging (DTI) sequence (TR = 7.5 s, minimum TE, FOV = 25.6 cm, 128 × 128 matrix, slice thickness = 2 mm). Diffusion-sensitizing gradients were applied at a b-value of 1000 s/mm^2^ along 30 non-collinear directions.

Preprocessing was done using the FMRIB Software Library (FSL 5.0.8) (Smith et al., 2004) and MATLAB. A quality assurance step was performed, in which diffusion volumes affected by motion were rejected (Tymofiyeva et al., 2012) and remaining images were corrected for eddy current distortions, affine head motion, and b-vector rotation. The DTI reconstruction, deterministic whole-brain streamline fiber tractography and visualization were performed using the Diffusion Toolkit (Wang et al., 2007). Fiber Assignment by Continuous Tracking (FACT) was chosen with one seed per voxel and a standard threshold angle of 35°. The entire diffusion-weighted volume was used as the mask image.

Cerebral segmentation into 90 regions of interest (ROIs) was performed in each individual’s DTI space using the Automated Anatomical Labeling (AAL) atlas (Tzourio-Mazoyer et al., 2002) and intermediate registration to T1-weighted images in MNI space. The ROIs were dilated by one voxel and served as network nodes. To define the connection (edge) between any two nodes, the average FA value within voxels intersected by the streamlines connecting those nodes was used. Network *centrality* of a node was calculated as the sum total of connection weights between that node and the rest of the nodes. Network visualization was performed using Gephi, an open-source network visualization software package (Bastian et al., 2009).

### Statistical Analyses

The statistical analyses were performed using IBM SPSS Statistics software (version 25). Continuous variables were summarized using mean, median, and standard deviations. Linear regressions were performed using smartphone dependence as a dependent variable and mesolimbic node centralities and sex as independent variables. Bivariate correlations were evaluated using Pearson’s correlation coefficient. The smartphone dependence was compared between female and male subjects using a two-sample *t*-test with equal variances. *P*-values were not adjusted for multiple comparisons due to the exploratory nature of the study.

## Results

**FIGURE 1:**
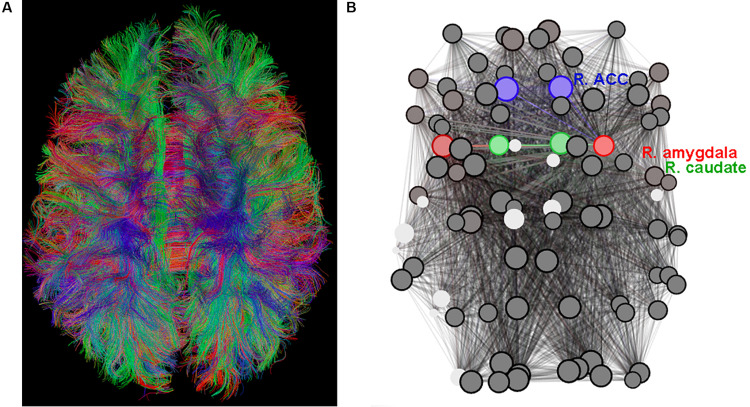
**(A)** Diffusion tensor imaging (DTI)-based whole-brain tractogram in an adolescent. **(B)** The corresponding graph (set of nodes and edges) depicting the connectome with amygdala nodes highlighted in red, caudate nodes highlighted in green (include Nucleus Accumbens), and anterior cingulate cortex (ACC) nodes highlighted in blue.

The MRI scan was well-tolerated by all subjects. A typical result of the whole-brain tractography reconstruction is shown in Figure 1A and a graph (brain network) is shown in Figure 1B.

Summary statistics are presented in Table 1. Our results revealed a significant association between the node centrality of the right amygdala and smartphone dependence SAS-SV score (Pearson’s r = 0.56; *p* = 0.01). The linear regression analysis with the smartphone dependence as the dependent variable and the right amygdala node centrality as an independent variable resulted in *p* = 0.004, after including sex as a predictor in the linear regression (Table 2). Centralities of other nodes were not significantly correlated with smartphone dependence (Table 2).

**TABLE 1:** Summary statistics for the studied continuous variables.

	*n*	Mean	Standard deviation	Min	Median	Max
Age, years	19	16.3	1.185	13.84	16.12	18.04
Phone dependence (SAS-SV score)	19	2.936	0.917	1.4	2.889	5
Sleep problems (ISI)	19	10.58	4.67	3	11	18
Depressive symptoms (RADS-2 t-score)	19	64.11	16.15	36	66	98
Amygdala L node centrality	19	0.289	0.045	0.215	0.295	0.364
Amygdala R node centrality	19	0.332	0.044	0.254	0.331	0.43
Caudate L node centrality	19	0.365	0.035	0.273	0.367	0.412
Caudate R node centrality	19	0.38	0.035	0.298	0.383	0.437
ACC L node centrality	19	0.38	0.027	0.323	0.377	0.428
ACC R node centrality	19	0.386	0.025	0.324	0.385	0.429

*SAS-SV, Smartphone Addiction Scale - Short Version; ISI, Insomnia Severity Index; RADS-2, Reynolds Adolescent Depression Scale-2; L, left; R, right; ACC, anterior cingulate cortex; n, number of participants.*

**TABLE 2:** Results of the univariate and multivariate linear regressions with smartphone dependence SAS-SV score as the outcome variable.

	Univariate model			Sex adjusted model		
	Coefficient	SE	*p* value	Coefficient	SE	*p* value
Sleep problems (ISI)	0.095	0.042	0.035	0.07	0.04	0.103
Depressive symptoms (RADS-2 t-score)	0.021	0.013	0.118	0.009	0.013	0.511
Amygdala L node centrality	8.566	4.479	0.073	7.039	4.017	0.099
Amygdala R node centrality	11.72	4.213	0.013	11.42	3.424	0.004
Caudate L node centrality	4.218	6.323	0.514	1.118	5.761	0.849
Caudate R node centrality	4.893	6.304	0.448	4.668	5.503	0.409
ACC L node centrality	13.61	7.607	0.091	13.37	6.458	0.055
ACC R node centrality	14.29	8.187	0.099	10.99	7.454	0.16

*Coefficient and SE – coefficients and standard errors in the linear regression models with and without adjusting for the sex effect, where smartphone dependence SAS-SV score is the outcome variable in the models. *P* value – *p*-values for testing the null hypothesis that the regression coefficients equal 0 using the Wald type tests. SAS-SV, Smartphone Addiction Scale - Short Version; ISI, Insomnia Severity Index; RADS-2, Reynolds Adolescent Depression Scale-2; L, left; R, right; ACC, anterior cingulate cortex.*

A positive correlation was observed between smartphone dependence measured using SAS-SV and sleep problems measured using ISI (Pearson’s r = 0.49; *p* = 0.04), and between sleep problems and depressive symptoms measured using RADS-2 (Pearson’s r = 0.67; *p* = 0.002).

Females showed a significantly higher phone dependence measured by SAS-SV (Cohen’s d = −1.18, *p* = 0.03).

## Discussion

In this study, we utilized diffusion MRI and connectomics to investigate the neural correlates of smartphone dependence in adolescents. The results of the present study were consistent with our hypothesis regarding involvement of the mesolimbic network in smartphone dependence. Specifically, the node centrality of the right amygdala (but not of the other nodes, NAcc and ACC) was positively correlated with smartphone dependence. This result is consistent with previous literature in adult users of digital technologies, in particular social media: three MRI studies of social network site (SNS) users all support the key role of amygdala in addictive behavior with regard to social media use. Specifically, Turel et al. showed that Facebook users with addiction-like symptoms have a hyperactive amygdala-striatal system (Turel et al., 2014). Using voxel-based morphometry (VBM) applied to structural MRI scans of 20 SNS users, He et al. showed that SNS addiction is associated with a presumably more efficient impulsive brain system, manifested through reduced gray matter volumes in the amygdala bilaterally (He et al., 2017a). The same group of authors showed similar results in 50 university students and concluded that excess social media use is associated with gray matter volume reduction in the bilateral amygdala (He et al., 2017b). This finding makes SNS addiction similar to other addictions (substance use, gambling, etc.). However, in contrast to other addictions in which the ACC is impaired and fails to support the needed inhibition, this brain region was not affected in their sample (He et al., 2017b). Similarly, we did not observe correlation of smartphone dependence scores with the node centrality of ACC and NAcc in our study. The heightened network centrality of the right amygdala that we observed indicates higher structural connectivity (meaning, e.g., more abundant white matter fibers and/or their myelination) of the amygdala with other brain regions. By linking environmental cues to reward systems in the striatum, the amygdala may become over-sensitized with repetitive smartphone use and the associated strong rewards, which can lead to a constant desire to enact the addictive behavior (Robinson and Berridge, 2001).

Apart from the neural correlates, we have also explored associations between smartphone dependence and sleep problems, and between sleep problems and depressive symptoms in our sample. We observed moderate/strong positive correlations in both cases, which may point at one potential mediating link between excessive smartphone use and the recently reported increase in mental health problems among adolescents: namely, through the sleep problems caused by excessive screen time.

As mentioned above, a recent study reported increases in depressive symptoms, suicide-related outcomes, and suicide rates among United States adolescents (Twenge et al., 2018). Twenge et al. (2018) investigated different factors, such as television watching, homework, and cyclical economic factors, however, they could only link the observed increases in mental health issues to greater new media screen time. Adolescents who spent more time on screen activities (including social media and electronic devices such as smartphones) were significantly more likely to report mental health issues, and adolescents who spent more time on non-screen activities (in-person social interaction, sports/exercise, homework, etc.) were less likely to report such issues. Specifically, among those who used electronic devices five or more hours a day, 48% had at least one suicide-related outcome. Other factors, such as watching TV, homework, and economic factors such as unemployment and the Dow Jones Index, could not explain the rise of the mental health problems. The timing of the increase in mental health issues, beginning around 2011–2012, is also worth noting. About half of Americans used smartphones by late 2012. By 2015, 92% of teens and young adults owned a smartphone (Smith, 2017). Thus, smartphones were used by the majority of teens the year that depressive symptoms began to increase and by nearly all teens when depressive symptoms peaked.

While reverse causality and other factors cannot be excluded, evidence from the study by Twenge et al. (2018) supports the model in which increased screen time leads to increased depressive symptoms, especially in females. Additionally, studies of adult users of social media provide evidence that screen time may lead to depressive symptoms and not the other way around. One longitudinal study showed that more frequent use of Facebook led to more negative mood later, whereas negative mood did not increase Facebook use (Kross et al., 2013). Another longitudinal study that used an annual assessment for three years found that Facebook use decreased psychological well-being among 5,208 adults, whereas in-person social interaction increased it (Shakya and Christakis, 2017). In another, experimental study, 1,095 adults were randomly assigned to either continue their usual Facebook use or to give it up for a week. Participants who gave up Facebook had fewer depressive symptoms at the end of the week than those who continued using Facebook, suggesting that Facebook use leads to depressed mood (Tromholt, 2016). The results of these studies indicate that there is likely at least some partial causality of depression from social media use.

One potential mediating link between excessive smartphone use and mental health problems are the sleep problems caused by excessive screen time. Our results obtained in adolescents point at this possibility as well. Positive correlations between the smartphone addiction and sleep quality scores were also found in 319 university students (Demirci et al., 2015). In the study by Hu et al. (2017) control subjects reported an average sleep time of 7.4 h per night, whereas young adults with smartphone dependence slept on average 5.2 h per night. In a study by Orzech et al. (2016) 254 first-year University students tracked their sleep through daily online diaries and provided digital media use data in 15-min blocks for 2 h before bedtime on nine occasions. A longer duration of digital media use was associated with reduced total sleep time and later bedtime. Mobile phone use by adolescents after lights out was investigated in a prospective cohort study with a one-year follow-up (Van den Bulck, 2007). The study concluded that using the phone after going to bed leads to increasing sleep problems (measured as increased levels of tiredness one year later).

The sleep problems associated with smartphone use may in turn lead to the reported increases in depressive symptoms, as sleep has often been causally implicated in depression (Baglioni et al., 2011; Jones and Benca, 2015). This is also supported by the second part of our exploratory analysis. Although it had been long considered that sleep problems are merely symptoms of depression, experimental evidence points at a more complex, bi-directional relationship (Krystal, 2012). Sleep problems, such as insomnia or hypersomnia, is one of the criteria for the clinical diagnosis of major depressive disorder (MDD) (APA, 2013). Having an MDD diagnosis is often associated with one or several sleep difficulties such as difficulty falling asleep, difficulty staying asleep, daytime sleepiness, insufficient sleep quality, and nightmares. Importantly, people with sleep problems are approximately ten times more likely to have MDD and “having insomnia at one point in time significantly increases the risk for the subsequent development of new onset MDD” (Krystal, 2012). Moreover, numerous studies including prospective studies in adults and studies in children and adolescents have linked sleep problems to suicidality (Krystal, 2012). For example, Gangwisch et al. (2010) examined data from a large epidemiologic data set gathered from adolescents (grades 7 to 12) in the United States. The analysis showed that adolescents with parental set bedtimes of midnight or later relative to those with bedtimes set at 10:00 pm or earlier, were significantly more likely to suffer from depression or suicidal ideation. The authors also found that this association was mediated by total sleep time; thus, those with earlier bedtimes reported sleeping more and were less likely to be depressed or experience suicidal ideation.

Furthermore, one previous study points at the mediating role of poor sleep in the relationship between the intensity of usage of information and communication technology and poor teen health, including depressive symptoms. Punamäki et al. (2007) studied 7,292 Finns aged 12, 14, 16, and 18 years. First, the results showed gendered use of technology: boys played digital games and surfed the Internet more often than girls, whereas girls had preference for mobile phone communication (Punamäki et al., 2007). Structural equation model analyses supported the mediating hypothesis of sleep: intensive use of technology was associated with poor perceived health only when it negatively affected sleep, which in turn correlated with tiredness during the day. The mediating links were also gendered, especially among older adolescents. Intensive phone use formed a risk for girls’, and intensive computer use formed a risk for boys’ poor perceived health.

Finally, the results of our study provide additional support for the observation that smartphone dependence is more common in females. As described above, Twenge et al. recently reported that depressive symptoms, suicide-related outcomes, and suicide deaths among adolescents all rose during the 2010s after being stable or declining (Twenge et al., 2018). The increase in depressive symptoms was driven almost exclusively by females. Between 2009/2010 and 2015, 58% more females scored high in depressive symptoms (16.74% in 2010, 26.40% in 2015) and 14% more reported at least one suicide-related outcome (Twenge et al., 2018). At the same time, literature reviews report that virtually all the studies indicate that females have higher levels of smartphone dependence and problematic use than males (De-Sola Gutiérrez et al., 2016). For example, in two studies of 342 adolescents (Randler et al., 2016) and 319 university students (Demirci et al., 2015), females were shown to be more likely to be addicted to smartphones. In a large Finnish study of 7,292 adolescents mobile phone usage was also more intensive in girls (Punamäki et al., 2007). A potential explanation for the observed sex differences is that women have socially related motives for using phones compared with men who have more utilitarian and/or entertainment motives (Roberts et al., 2014).

**FIGURE 2:**
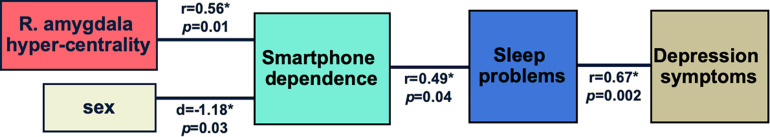
Proposed model of how smartphone dependence is linked to aberrations in brain networks, sex, sleep problems, and depressive symptoms. The results obtained in this study are shown: r stands for Pearson’s correlation coefficient, d - for Cohen’s d. *indicates statistical significance at 0.05 level. It needs to be emphasized that due to the limited sample size we did not test this model as a whole but rather explored individual bivariate correlations.

Here, based on prior published research and our findings, we propose a model of how smartphone use can be linked to aberrations in brain networks, sex, sleep problems, and depressive symptoms in adolescents (Figure 2). In summary, according to this model increased smartphone dependence is associated with being female and with heightened network centrality of the amygdala, and leads to increased sleep problems and depressive symptoms in adolescents. It needs to be emphasized that due to the limited sample size we did not test this model as a whole but rather explored individual bivariate correlations.

Previous findings of abnormal brain structure in SNS addiction (He et al., 2017a,b), as well as our results, possibly point to predisposing factors that, in the case of this study, contribute to both hyperconnected amygdala and the propensity for smartphone addiction. Alternatively, these findings could reflect the deleterious effects of excessive use of digital technologies on neuronal structure.

The results of our study must be interpreted in light of its limitations. Our inability to determine the causality is due to the cross-sectional study design, which is the main limitation of the present study. Another limitation is the small sample size included in this study. In spite of these limitations, the obtained knowledge can contribute to our understanding of the brain network disruptions associated with smartphone dependence in adolescents.

## Conclusion

In conclusion, our cross-sectional MRI study in a community sample of 19 adolescents showed the following positive correlations: between a brain network centrality measure of the right amygdala and self-reported smartphone dependence, between smartphone dependence and sleep problems, and between sleep problems and depressive symptoms. Female adolescents in our study showed a higher phone dependence compared to male teens. Based on these results and prior published research, we propose a model of how smartphone dependence can be linked to aberrations in brain networks, biological sex, sleep disturbances, and depression in teens. The results of our study and the proposed model (once tested in large-scale studies) can aid in the development of effective and empowering interventions to help young people regulate their behavior with respect to modern technologies and, potentially, prevent adolescent depression and suicide.

## Data Availability Statement

The datasets presented in this article are not readily available because although the final MRI datasets will be stripped of identifiers prior to release for sharing, we believe that there remains the possibility of deductive disclosure of subjects with unusual characteristics. We will therefore make the data and associated documentation available to users only under a data-sharing agreement that provides for the following: (1) a commitment to using the data only for research purposes and not to identify any individual participant; (2) a commitment to securing the data using appropriate computer technology; and (3) a commitment to destroying or returning the data after analyses are completed. Requests to access the datasets should be directed to Olga Tymofiyeva, olga.tymofiyeva@ucsf.edu.

## Ethics Statement

The studies involving human participants were reviewed and approved by Institutional Review Board (IRB) of the University of California, San Francisco. Written informed consent to participate in this study was provided by the participants’ legal guardian/next of kin.

## Author Contributions

OT, RK, C-YH, EH, MR, TY, and DX designed the study. OT, JY, EH, and DX collected the data. OT, JY, RK, and C-YH processed and analyzed the data. OT, JY, RK, C-YH, EH, MR, NJ, JM, TY, and DX wrote the manuscript. All authors contributed to the article and approved the submitted version.

## Conflict of Interest

The authors declare that the research was conducted in the absence of any commercial or financial relationships that could be construed as a potential conflict of interest.
